# Relationship Between Eyelid Pressure and Lacrimal Status in Mild Facial Nerve Palsy

**DOI:** 10.7759/cureus.12830

**Published:** 2021-01-20

**Authors:** Patricia Ann L Lee, Aric Vaidya, Shinjiro Kono, Hirohiko Kakizaki, Yasuhiro Takahashi

**Affiliations:** 1 Oculoplastic, Orbital and Lacrimal Surgery, Aichi Medical University Hospital, Aichi, JPN; 2 Ophthalmology, Rapti Eye Hospital, Dang, NPL

**Keywords:** facial nerve palsy, eyelid pressure, lacrimal status

## Abstract

Purpose: Facial nerve palsy is frequently associated with both epiphora and dry eye, and orbicularis oculi muscle weakness or paralysis is the main cause of these symptoms. Eyelid pressure is a quantitatively measurable parameter for evaluating the tone and function of the orbicularis oculi muscle. The aim of this study was to examine the relationship between eyelid pressure and lacrimal status in patients with mild facial nerve palsy.

Methods: This prospective, interventional study included 10 patients with unilateral facial nerve palsy. The severity of facial nerve palsy was determined using the CADS scale (cornea, static asymmetry, dynamic function, and synkinesis). Eyelid pressure was measured using a blepharo-tensiometer. Lacrimal status was quantified through tear meniscus height (TMH), clinical assessment of meibomian gland dysfunction (MGD) (eyelid margin abnormalities, Marx line, meibum expression, and loss of meibomian glands), corneal fluorescein staining, tear break-up time, and Schirmer test I results.

Results: All 10 patients suffered from mild facial nerve palsy without eyelid ectropion or entropion, or gustatory epiphora. Lower eyelid pressure during forceful eye closure was significantly decreased in affected eyes (P = 0.007), but upper eyelid pressure during forceful eye closure and static upper and lower eyelid pressure were not significantly different between the affected and unaffected sides (P > 0.050). The TMH, MGD, and dry eye measurements showed no significant difference between the affected and unaffected eyes (P > 0.050).

Conclusions: Mild facial nerve palsy is associated with decreased lower eyelid pressure during forceful eye closure. However, no other differences in upper eyelid pressure during forceful eye closure, static eyelid pressure, TMH, MGD, or dry eye disease symptoms were noted. These results imply that eyelid pressure slightly decreases due to mild facial nerve palsy, but this change may be clinically negligible.

## Introduction

Facial nerve palsy is frequently associated with both epiphora and dry eye [1], and orbicularis oculi muscle (OOM) weakness or paralysis is the usual culprit behind these symptoms [2-5]. Poor muscle tone can impair proper lacrimal pump function, resulting in epiphora [2,3]. Traditionally, eyelid margin has been considered the area of eyelid most in contact with the cornea and is held responsible for spreading the tear film across the entire ocular surface [4,5]. Poor eyelid margin apposition to the globe due to poor muscle tone can create an irregular tear surface, which can promote dry eye disease [5]. Moreover, the muscle of Riolan, which is part of OOM along the eyelid margin, controls the secretion of tear lipids [6]. Similarly, poor OOM function impairs the muscle of Riolan, causing meibomian gland dysfunction (MGD) [6,7].

Eyelid pressure is a quantitatively measurable parameter that can be used to evaluate OOM tone and function. While several measurement methods for eyelid tension have been proposed [8-10], Sakai et al. introduced an outstanding method that directly measures the eyelid pressure on the ocular surface using a tactile pressure sensor [11].

None of the previous studies examined the relationship between eyelid pressure and lacrimal status in patients with facial nerve palsy. We examined this relationship using a tactile pressure sensor. Only those patients with mild facial nerve palsy were included in this study because eyelid pressure and tear meniscus height (TMH) cannot be measured accurately in patients with paralytic eyelid ectropion or entropion, and also, the relationship between eyelid pressure and TMH cannot be validated in patients with gustatory epiphora.

## Materials and methods

This was a prospective, interventional study involving Japanese patients with mild unilateral facial nerve palsy who underwent surgical correction for various related symptoms from November 2016 to March 2017. None of the patients had undergone previous eyelid surgery nor any history of contact lens use. All patients showed a patent lacrimal drainage system confirmed by lacrimal syringing.

All measurements and assessments were performed by one of the authors (Y.T.) before surgical correction. The severity of facial nerve palsy was assessed according to the CADS (cornea, static asymmetry, dynamic function, and synkinesis) grading scale reported by Malhotra et al. [12]. As corneal staining was evaluated using another grading system described later, the cornea (C) grading system was not used in this study. Static asymmetry (A) was assessed based on severity of brow ptosis (0, absent; 1, mild; 2, severe), ectropion (0, absent; 1, mild; 2, significant), eyelid retraction (0, absence; 1, mild; 2, severe), and lid margin-to-brow distance (1, >5 mm shorter than contralateral side; 2, ≤20 mm). Dynamic function (D) was evaluated as follows: 0, no blink lagophthalmos; 1, lagophthalmos on blink <5 mm or the presence of reduced brow elevation; 2, lagophthalmos on blink ≥5 mm, lagophthalmos on gentle closure ≤5 mm, or none or twitch of brow elevation; 3, lagophthalmos on gentle closure ≤5 mm or lagophthalmos on forced closure > 5 mm. Synkinesis (S) was graded as follows: 0, absent; 1, mild eyelid closure when smiling, speaking, and/or eating, or mild gustatory epiphora; 2, significant eyelid closure when smiling, speaking, and/or eating, or bothersome gustatory epiphora.

Eyelid pressure was measured using a tactile pressure sensor (DigiTacts Single Point Sensors; Pressure Profile Systems, Inc, Los Angeles, CA) according to a previous study conducted by Sakai et al. [11]. The sensor tip was not waterproof and thus, covered with a 0.03-mm-thick disposable waterproof polyurethane cap (Okamoto Industries, Inc, Tokyo, Japan). A new cap was used for each patient to prevent the spread of infection. The pressure sensor was connected to a personal computer, which took measurements automatically every 0.03 seconds.

**Figure 1:**
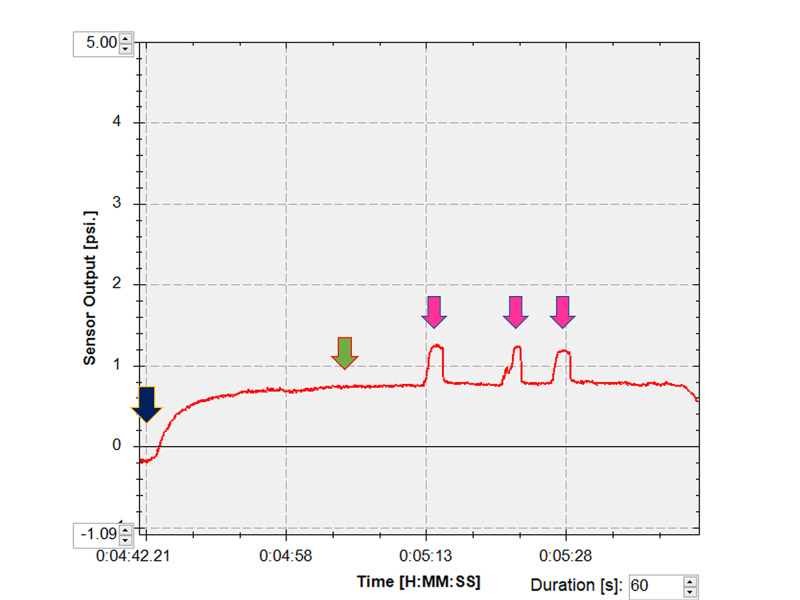
Measurement of eyelid pressure After the pressure sensor was inserted into the conjunctival fornix (blue arrow), the patient kept his/her eyes open until the measured pressure became a plateau, which was continued for five seconds (green arrow). Then, the patient closed his/her eyes forcefully three times (pink arrows). psi, pounds per square inch.

Following the instillation of topical 0.4% oxybuprocaine (Santen, Osaka, Japan), each patient was fitted with a sterile disposable soft contact lens (-0.5 diopters, Focus DAILIES; CIBA Vision, Duluth, GA) to protect against corneal abrasions. The pressure sensor was then inserted into the center of the upper or lower conjunctival fornix. The patients were asked to keep their eyes open until the measured pressures reached a plateau phase that was maintained for five seconds (Figure 1). They were then directed to close their eyes forcefully three times. The mean maximum pressure during forceful eyelid closure was calculated (Figure 1). Eyelid pressure at the plateau phase and mean maximum pressure were used for statistical analysis.

TMH was measured on the sagittal plane through the center of the upper and lower eyelids using optical coherence tomography (RS-3000, NIDEK CO., LTD, Aichi, Japan) [13,14]. The dedicated attachment was used to observe the anterior segment of the eyes.

MGD was assessed through four criteria: the presence or absence of eyelid abnormalities; the position of Marx line; the quality and ease by which meibum is expressed and status of the meibomian glands, as based on our previous studies [6,15].

The presence or absence of eyelid abnormalities (irregular lid margin, vascular engorgement, plugged meibomian orifices) was expressed using a binary system (a dummy variable; 0 = absence, 1 = presence), and total sum scores were calculated in each upper and lower eyelid for statistical analysis. The position of Marx line was determined by fluorescein staining of the ocular surface. At the time of fluorescein staining, strict attention was paid not to increase the subjects’ tear volumes. A drop of physiological saline solution was put on a fluorescein test strip. After the excess saline solution was shaken off the strip, the strip was gently touched to the center of the lower eyelid margin. The resulting stained line along the affected lid was examined by slit-lamp biomicroscopy after several blinks. The grading scale reported by Yamaguchi et al. was used to determine the Marx line score: grade 0, the line runs entirely along the conjunctival side of the meibomian gland orifices; grade 1, parts of the line touch the meibomian orifices; grade 2, the line runs through the meibomian orifices; and grade 3, the line runs along the eyelid margin side of the meibomian orifices [16]. The lid was further divided into three segments (the outer third, middle third, and inner third) and the position of Marx line was evaluated in each; the maximum possible score for each lid was 9. The quality and ease by which meibum was expressed were graded as follows: grade 0, easy expression of clear meibum with mild eyelid compression; grade 1, cloudy meibum expression with mild compression; grade 2, cloudy meibum expression with moderate compression; grade 3, toothpaste-like meibum expression with more than moderate compression; and grade 4, no expression even with hard compression [6,15]. The status of meibomian glands in the affected lower lid was evaluated using mobile pen-shaped meibography (Meibom Pen, Japan Focus Co., Ltd., Tokyo, Japan). The grading scale previously reported by Arita et al. was used: grade 0, no loss of meibomian glands; grade 1, area loss less than one-third of the total meibomian gland area; grade 2, area loss between one-third and two-thirds of total meibomian gland area; and grade 3, area loss more than two-thirds of total meibomian gland area [17].

The area (A) and density (D) classification of corneal fluorescein staining, tear break-up time (TBUT), and Schirmer test I were also used to assess the severity of dry eye. The AD classification was graded using the scale reported by Miyata et al. [18]. The A was classified as follows: grade 0, no punctate staining; grade 1, the staining involving less than one-third of the cornea; grade 2, the staining involving one-third to two-thirds of the cornea; and grade 3, the staining involving more than two-thirds of the cornea. The D was classified as follows: grade 0, no punctate staining; grade 1, sparse density; grade 2, moderate density; and grade 3, high density, and overlapped lesions. The TBUT was determined by fluorescein staining of the ocular surface. The time just after eye-opening to the first appearance of a dry spot on the cornea was measured. Schirmer test I was performed without anesthesia as follows: a Schirmer test strip was placed in the lower conjunctival sac without touching the cornea, and the length of the wet portion after five minutes was measured.

The patient’s age and measurement results were expressed as means ± standard deviations. The measurements were compared between the affected and unaffected sides using the Mann-Whitney U test. All statistical analyses were performed using SPSS™ ver. 26 software (IBM Japan, Tokyo, Japan). A P-value of <0.05 was considered statistically significant.

## Results

Patient demographic data and measurement results are shown in Tables 1 and 2. This study included 10 patients with unilateral facial nerve palsy (four males and six females; six right and four left; mean age, 75.3 ± 5.6 years; range, 66 to 81 years). Bell’s palsy was the cause of facial nerve palsy in all patients. The mean scores of the A, D, S grading scales of facial nerve palsy were 1.3 ± 0.5, 0, and 0, respectively. The mean duration between the onset and the time of measurements was 12.8 ± 10.9 years (range, 1 to 30 years).

**Table 1:** Patient demographic data

Number of patients	10
Sex (male/female)	4/6
Side (right/left)	6/4
Age (years)	75.3 ± 5.6
CADS score	
A	1.3 ± 0.5
D	0
S	0
Duration from onset (years)	12.8 ± 10.9

**Table 2:** Comparison of measurement results between affected and unaffected sides TMH, tear meniscus height; TBUT, tear break-up time.

	Affected side	Unaffected side	P-value
Maximum eyelid pressure (psi)			
Upper	1.09 ± 0.16	1.27 ± 0.29	0.143
Lower	1.15 ± 0.16	1.43 ± 0.25	0.007
Eyelid pressure at plateau phase (psi)			
Upper	0.89 ± 0.13	0.92 ± 0.20	0.853
Lower	0.83 ± 0.16	0.89 ± 0.22	0.481
TMH (μm)			
Upper	242.7 ± 96.6	207.0 ± 97.4	0.481
Lower	274.5 ± 101.1	244.2 ± 51.8	0.436
Eyelid abnormality			
Upper	1.5 ± 1.1	1.1 ± 1.0	0.436
Lower	1.5 ± 0.8	1.4 ± 1.0	0.739
Marx line			
Upper	7.7 ± 1.6	6.6 ± 1.5	0.190
Lower	7.9 ± 1.5	7.2 ± 1.6	0.353
Meibum expression			
Upper	2.7 ± 1.3	2.0 ± 1.5	0.353
Lower	2.5 ± 1.3	2.1 ± 1.3	0.579
Loss of meibomian glands			
Upper	1.5 ± 1.0	1.5 ± 1.0	1.000
Lower	1.1 ± 0.9	0.8 ± 1.0	0.393
AD classification			
A	0.8 ± 0.8	0.3 ± 0.7	0.165
D	1.2 ± 1.3	0.3 ± 0.7	0.105
TBUT (second)	1.6 ± 2.1	1.0 ± 1.1	0.739
Schirmer test I (mm)	9.1 ± 8.3	6.1 ± 4.5	0.353

The mean maximum eyelid pressure in the lower lid was significantly lower on the affected side compared to the unaffected side (P = 0.007), although the mean maximum eyelid pressure in the upper lid showed no significant difference between the affected and unaffected sides (P = 0.143). The eyelid pressure at the plateau phase showed no difference between the affected and unaffected sides (upper, P = 0.853; lower, P = 0.481).

**Figure 2:**
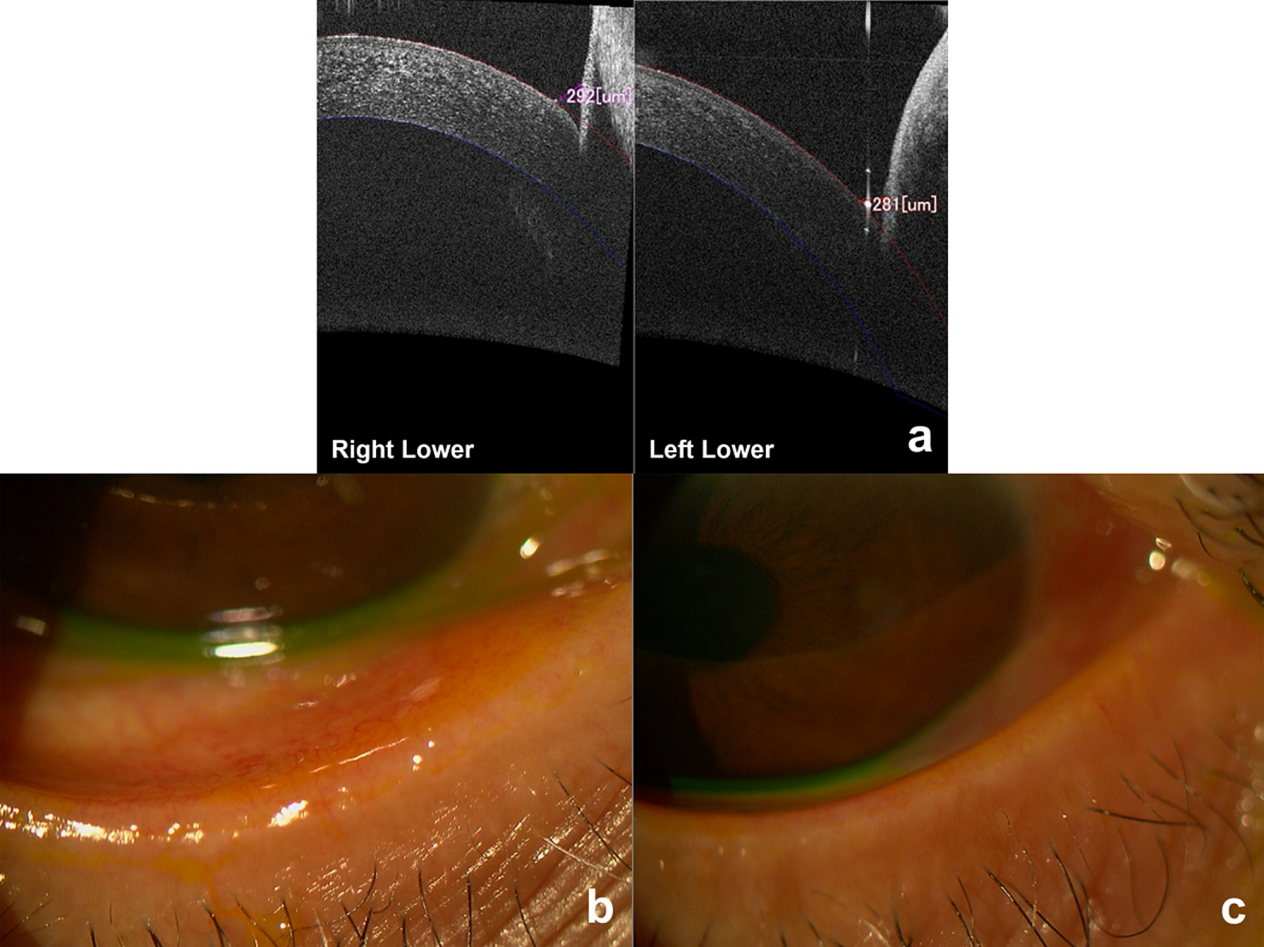
Findings of a representative case. (a) TMH in the affected side (right lower) was similar to that in the unaffected side (left lower). Meibomian gland dysfunction (b) right side (affected side) and (c) left side (unaffected side). Vascular engorgement, plugged meibomian gland orifices, and advancement of the mucocutaneous junction are present on both sides. TMH: Tear meniscus height

There was also no significant difference in TMH between the affected and unaffected sides (upper, P = 0.481; lower, P = 0.436) (Figure 2a). All the criteria used in the evaluation of MGD and dry eye showed no significant difference between the affected and unaffected sides (P > 0.050) (Figures 2b, 2c), as well.

## Discussion

Our study found that the lower eyelid pressure during forceful eye closure was significantly decreased in the setting of facial nerve palsy. However, we also found that the static upper and lower eyelid pressure and the upper eyelid pressure during forceful eye closure were not significantly decreased in the affected eyes. These findings seem to indicate that the eyelid pressure and consequently, the eyelid tone and function, are largely maintained in patients with mild facial nerve palsy.

The parameters, such as TMH, MGD, and dry eye measurements, also showed no significant difference between the affected and unaffected eyes. While the patients with facial nerve palsy occasionally complain of dry eye symptoms [4-6], these symptoms tend to be unrelated to the changes in TMH, meibomian gland function, and dry eye conditions. As mentioned above, the eyelid pressure was largely maintained in mild facial nerve palsy, which may be helpful to maintain the lacrimal status as well. Another possible reason was that all the patients were old (mean age, 75.3 ± 5.6 years; range, 66 to 81 years). The old patients usually show MGD and dry eye [5], as shown in the unaffected side in this study, which might have minimized the side-related difference in our study. Therefore, analyzing patients with moderate and severe palsy or young patients may give different results. Also, future iterations of this study can benefit from a similar study on a larger number of patients, correlating the severity of facial nerve palsy with the signs and symptoms, examining the degree of corneal exposure and its relationship with dry eye symptoms, and by measuring the eyelid pressure during normal blink conditions.

Conventionally, the House-Brackman grading system, which assesses the facial muscle movement and tone, has been used to evaluate the severity of facial nerve palsy [19]. However, ophthalmologic assessment in this system is limited to whether there is a complete or incomplete eyelid closure and thus, provides an inaccurate picture of eye involvement. The CADS scale [12], combined with standard dry eye evaluation tests, can measure the eye sequelae more effectively.

A previous study that used a tactile pressure sensor showed opposite results, wherein the lower eyelid pressure in patients with functional epiphora was lower than in normal controls [20]. However, this previous study did not compare lacrimal status between patients with functional epiphora and normal controls [20].

When patients with mild facial nerve palsy show functional epiphora, this can be resolved by tightening of the lower eyelid with a lateral tarsal strip procedure [21].

Our study was limited by several factors. First, as mentioned above, our study included a small number of patients with mild facial nerve palsy. A larger number of subjects would have given much clearer results. Second, the inclusion of only Japanese patients was another limitation as the periocular anatomy has racial differences [22]. Therefore, the results of this study may not apply to other races. Third, all the examinations were performed by a single clinician, which may cause an examiner bias. Fourth, we did not examine for the presence or absence of Bell’s phenomenon. However, as none of the patients showed lagophthalmos (D score = 0), the data on Bell’s phenomenon may not be significant for this study.

## Conclusions

In conclusion, our study found that the lower eyelid pressure during forceful eye closure was significantly decreased in mild facial nerve palsy. However, the static upper and lower eyelid pressure and the upper eyelid pressure during forceful eye closure were not significantly decreased in the affected eyes. The lacrimal status also showed no significant difference between the affected and unaffected eyes. Our results imply that the eyelid pressure slightly decreases due to mild facial nerve palsy, but this change may be clinically negligible.
